# Diagnostic and therapeutic approaches to acute prostatitis in dogs: a survey of Italian veterinary practitioners

**DOI:** 10.3389/fvets.2026.1774785

**Published:** 2026-04-17

**Authors:** Martina Gavezzoli, Francesca Fidanzio, Giulia Catellani, Alessandro Vetere, Andrea Corsini, Martina Fumeo, Antonella Volta, Maria Carmela Pisu, Francesco Di Ianni

**Affiliations:** 1 Department of Veterinary Science, University of Parma, Parma, Italy; 2 VRC-Centro di Referenza Veterinario, Turin, Italy

**Keywords:** andrology diagnosis, antibiotic resistance, CPSE, dogs prostate, prostatitis

## Abstract

Acute prostatitis is a clinically significant condition in dogs that requires accurate diagnosis and appropriate therapeutic management. According to International Society for Companion Animal Infectious Diseases guidelines, culture and sensitivity testing (CST) is strongly recommended, ideally using prostatic samples when feasible, and antimicrobial therapy should be based on culture results and administered for an adequate duration. This study aimed to evaluate current diagnostic and therapeutic approaches to acute prostatitis among Italian veterinary practitioners, with particular emphasis on culture and sensitivity testing (CST), antimicrobial therapy, and hormonal management. An anonymous observational survey consisting of 18 mandatory questions was distributed online to veterinarians across Italy (February 10–24, 2025). Responses (*n* = 306) were analyzed descriptively, and intergroup comparisons by facility type [small clinics (SC), large clinics (LC), and veterinary hospitals (VH)] were performed using chi-squared tests. Most respondents (93.1%) reported using a combination of clinical examination, bloodwork, urinalysis, and ultrasound for diagnosis. CST was performed in more than 50% of cases by 70% of VH, but only by 16% of SC. Urine was the most commonly used diagnostic matrix (54.9%), whereas samples of prostatic origin were used in only 20.9% of cases. Financial cost and turnaround time were identified as the main barriers to testing. Empirical antibiotic therapy was reported by 88.6% of veterinarians, most commonly amoxicillin-clavulanic acid (42%) and enrofloxacin (32%). However, only 7.2% of respondents prescribed antibiotics for the recommended duration of ≥4 weeks. Orchiectomy was performed in more than 50% of cases by only 19.6% of respondents. Hormonal therapy was used as a first-line treatment by 71% of practitioners. This study highlights significant deviations from current guidelines in both the diagnosis and treatment of canine prostatitis in general practice. Antimicrobial stewardship, the use of CST, and prostatic sampling should be improved to better align with current recommendations.

## Introduction

In intact male dogs, prostatic diseases are more common than in neutered ones (1). Prostatitis is the second most frequent prostatic disorder, with an incidence of 38% among all prostatic conditions; bacterial prostatitis, acute or chronic, accounts for approximately 40% of these cases (2). Acute prostatitis is characterized by a sudden and severe inflammation of the prostate, most often due to bacterial infection, and can progress to abscess formation (3). This condition is also associated with oxidative stress and altered antioxidant balance which may contribute to prostatic damage and exacerbate inflammation (4).

The predominant pathogens are Gram-negative bacteria of the Enterobacteriaceae family, especially *Escherichia coli*, although Gram-positive bacteria (*Staphylococcus* spp., *Streptococcus* spp.) have also been reported. *Brucella canis* can induce prostatitis experimentally, and *Mycoplasma* and *Ureaplasma* spp. may also be involved (2).

Clinical signs of acute prostatitis include fever, anorexia, lethargy, urethral discharge, and abdominal or perineal/prostatic pain. Rectal palpation is important for assessing prostate size, consistency, and pain, as inflamed prostates are often enlarged and irregular (5). Complete blood count and serum biochemistry typically reveal leukocytosis and neutrophilia with a left shift, often accompanied by elevated C-reactive protein (CRP) levels (6).

Abdominal ultrasonography (AUS) demonstrates an enlarged prostate with heterogeneous echotexture due to edema and inflammation, and it is also useful for identifying cysts or abscesses (7). In rare cases, a prostate biopsy is performed to exclude neoplasia. Measurement of Canine Prostate-Specific Esterase (CPSE) has been proposed as an additional diagnostic tool, as this cutoff is indicative of the presence of prostatic alterations in general. In particular, values >50 ng/ml are usually associated with benign prostatic hyperplasia and prostatitis (8–10).

Although urine CST is a common diagnostic method, it may yield negative results with the presence of a localized prostatic infection; discrepancies between bacterial isolates from urine and prostatic fluid have been reported (11).

Treatment of acute prostatitis involves antibiotics with adequate penetration through the blood–prostate barrier, such as fluoroquinolones, typically administered for a minimum of 4 weeks (12, 13).

Surgical castration remains the most effective long-term treatment for BPH, typically reducing prostate volume by up to 70% within weeks (14, 15). Hormonal therapies, such as finasteride and osaterone acetate, provide alternative management options (16–19), while chemical castration with deslorelin implants can also reduce prostatic volume and clinical signs, despite a possible transient flare-up (20). Recent studies have highlighted novel compounds which may offer additional therapeutic benefits in testosterone-induced BPH by modulating oxidative stress and Nrf2 signaling pathways, suggesting new directions for future research in prostatic disease management (21). Pain relief and anti-inflammatory therapy are also important for patient comfort (22).

Infected prostatic cysts could predispose to or complicate prostatitis, acting as bacterial reservoirs and requiring more aggressive or prolonged treatment. Ultrasound-guided aspiration of cysts >7–8 mm in diameter may help prevent secondary infections in dogs with cystic benign prostatic hyperplasia (5).

The prognosis is generally favorable when the disease is diagnosed and treated promptly (23).

This study aims to describe current diagnostic and therapeutic practices adopted by Italian veterinarians in the management of acute canine prostatitis, with particular attention to differences related to the workplace setting. In the absence of detailed, officially published guidelines for acute prostatitis, approaches to diagnosis, treatment, and follow-up may vary considerably. This survey, therefore, seeks to document existing clinical practices and highlight areas of variability that could support future efforts toward more consistent management strategies.

## Materials and methods

### Study design

An observational study was conducted to gather data on the diagnostic and therapeutic management of acute prostatitis in dogs. An anonymous questionnaire was designed using Google Forms and distributed to veterinarians practicing in different regions of Italy. The questionnaire was initially developed by two authors based on the study objectives and a review of the relevant literature. It was subsequently reviewed by the remaining authors, who contributed to refining content and structure. The revised version was pilot-tested by five veterinarians to assess clarity, feasibility, and completion time, to create a concise tool suitable for routine clinical practice. Minor modifications were implemented based on their feedback before final dissemination. The survey was shared through online forums and mailing lists of Provincial Veterinary Medical Associations, and remained available from February 10th to 24th, 2025. The questionnaire included 18 mandatory items, comprising both multiple-choice and open-ended questions (see Supplementary material S1).

The initial section of the survey collected information on the respondent's work setting, including geographical region, province, and type of veterinary facility. In accordance with national regulations, facilities were categorized as follows: small clinics (SC), primarily offering outpatient and primary care services; large clinics (LC), providing both primary and secondary care to in- and outpatients, without 24-h emergency coverage; and veterinary hospitals (VH), acting mainly as secondary and tertiary referral centers with 24-h emergency services.

Subsequent questions focused on the diagnostic approach to acute prostatitis, with options ranging from clinical assessment alone to combinations of diagnostic procedures such as hematology, biochemistry, CPSE, urinalysis, and AUS. Respondents were also asked whether culture and sensitivity testing (CST) was routinely performed; if not, further questions explored the reasons and challenges associated with its implementation. Additionally, information was collected regarding the most commonly used biological sample for diagnosis, whether urine, semen, or other.

The treatment section addressed therapeutic strategies, including the use of antibiotics, anti-inflammatory drugs, and hormonal therapy. Special attention was given to the class of antimicrobials selected for empirical treatment and the typical duration of therapy.

### Statistical analysis

A descriptive analysis was carried out, with results expressed as percentages. Comparative evaluations between different types of workplace settings were performed through *post-hoc* analysis using the Chi-squared test. Data were processed using commercially available statistical software (GraphPad Prism 9, San Diego, CA, United States), with statistical significance defined as *P* ≤ 0.05.

## Results

### Respondents

A total of 306 responses were collected through the questionnaire. The most represented regions were Lombardy (20.9%), Emilia-Romagna (19.9%), and Tuscany (16.3%; see Supplementary material S2). Among the participants, 181 (59.2%) work in SC, 75 (24.5%) in LC, and 50 (16.3%) in VH.

### Diagnosis

Overall, 93.1% of veterinarians diagnose acute prostatitis based on clinical evaluation, blood tests, urinalysis, and abdominal ultrasound. Serum measurement of CPSE during the diagnostic process is performed by 55 veterinarians (18%). The proportion of veterinarians who assess CPSE does not significantly differ among SC (17.7%), LC (16%), and VH (22%; *P* = 0.68).

**Figure 1:**
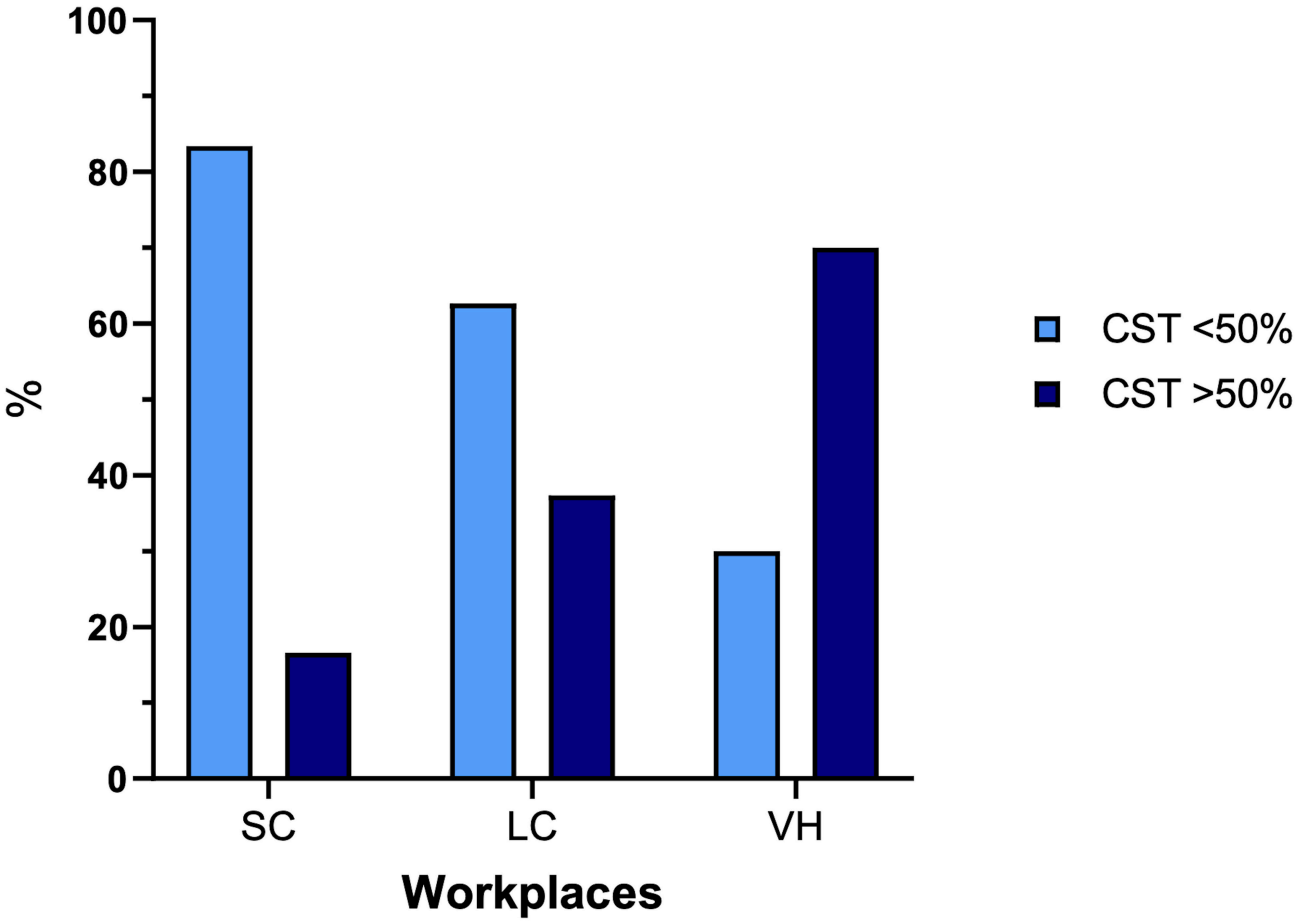
Percentage of responders performing CST in more and less than 50% of patients in different workplaces. SC, small clinics; LC, large clinics; VH, veterinary hospitals.

One-hundred and eight (51.6%) respondents performed CST in fewer than 25% of cases, 55 (18%) in 25%−50% of cases, 34 (11.1%) in 50%−75% of cases, and 59 (19.3%) in more than 75% of cases. The proportion of veterinarians who perform culture testing in over 50% of cases differs significantly between SC (16%), LC (37%), and VH (70%; *P* < 0.001; Figure 1).

**Figure 2:**
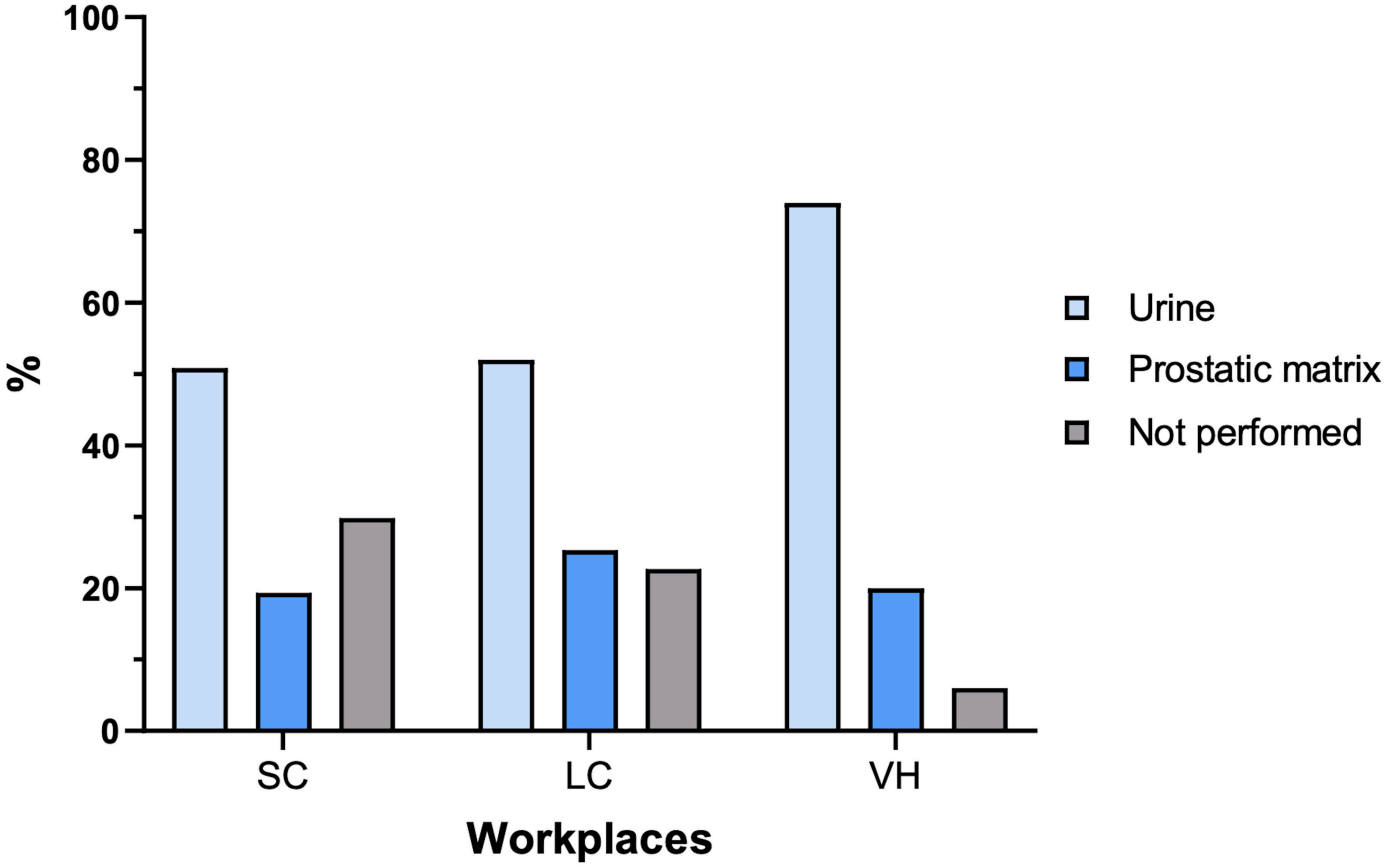
Percentage of biological samples used for CST in different workplaces. SC, small clinics; LC, large clinics; VH, veterinary hospitals.

In 20.9% of veterinarians (19.3% in SC, 25.3% in LC, and 20% in VH), the matrix employed for CST was of prostatic origin, including the prostatic fraction of seminal plasma, prostatic fluid obtained by ultrasound-guided catheterization, or material collected through fine-needle aspiration of the prostate. In 54.9% of cases urine is analyzed (50% in SC, 52% in LC, 74% in VH), with sterile collection performed in 45.8% of those. In 24.2% of cases, CST is never performed. These results show a statistically significant difference among the various types of facilities (*P* = 0.06), with 73% in SC, 22% in LC, and only 5% in VH not performing the examination (Figure 2).

The main reasons for not performing CST include financial constraints of the owners, long turnaround times for results, difficulties in sample collection (20.1%), perception that the test is unnecessary, and logistical challenges in shipping samples to external laboratories (Table 1).

**Table 1:** Reasons for not performing the culture and sensitivity testing in dogs with acute prostatitis among 306 veterinarians.

Possible answers (one or more)	Total number (%)	SC number (%)	LC number (%)	VH number (%)
Owner's financial problems	225 (55%)	124 (50%)	64 (62%)	37 (63%)
Difficulty in collecting the sample	81 (20%)	47 (20%)	21 (20%)	13 (22%)
Delayed result	64 (16%)	46 (19%)	14 (14%)	4 (7%)
I don't consider it necessary	29 (7%)	23 (9%)	2 (2%)	4 (7%)
Difficulty in sending the sample to the analysis laboratory	9 (2%)	6 (2%)	2 (2%)	1 (1%)
Total number of responses	408 (100%)	246 (100%)	103 (100%)	59 (100%)

SC, small clinics; LC, large clinics; VH, veterinary hospitals.

### Treatment

The combinations of first-line treatments used by veterinarians are reported in Table 2. Overall, 88.6% of veterinarians include antibiotics in their first-line treatment protocols (Table 2).

**Table 2:** First-choice treatment.

Treatments	Total number (%)	SC number (%)	LC number (%)	VH number (%)
Antibiotics, anti-inflammatories, and hormonal therapy	205 (67%)	127 (70%)	57 (76%)	21 (42%)
Antibiotics and hormonal therapy	45 (15%)	21 (12%)	13 (18%)	11 (22%)
Only antibiotics	21 (7%)	10 (6%)	3 (4%)	8 (16%)
Only hormonal therapy	12 (4%)	8 (4%)	1 (1%)	3 (6%)
Other treatments	19 (6%)	13 (7%)	1 (1%)	5 (10%)
Only anti-inflammatories	4 (1%)	2 (1%)	0	2 (4%)
Total number of responses	306 (100%)	181 (100%)	75 (100%)	50 (100%)

SC, small clinics; LC, large clinics; VH, veterinary hospitals.

The most prescribed antibiotics are amoxicillin-clavulanic acid (42%), enrofloxacin (32%), and marbofloxacin (12%; Table 3).

**Table 3:** Empirically prescribed antibiotics.

Antibiotic compound	Total number (%)	SC number (%)	LC number (%)	VH number (%)
Amoxicillin + clavulanic acid	129 (42%)	71 (39%)	35 (47%)	23 (46%)
Enrofloxacin	100 (32%)	62 (34.5%)	27 (36%)	11 (22%)
Marbofloxacin	38 (12%)	24 (13%)	10 (14%)	4 (8%)
No antibiotic therapy used	12 (4%)	7 (4%)	0	5 (10%)
Amoxicillin	8 (2.5%)	6 (3.5%)	1 (1%)	1 (2%)
Clindamycin	5 (2%)	3 (2%)	1 (1%)	1 (2%)
Ampicillin + sulbactam	4 (1%)	0%	0	4 (8%)
Cephalexin	3 (1%)	2 (1%)	1 (1%)	0
Cefazolin	2 (1%)	2 (1%)	0	0
Trimethoprim + sulfamethoxazole	2 (1%)	1 (0.5%)	0	1 (2%)
Cefadroxil	1 (0.5%)	1 (0.5%)	0	0
Metronidazole	1 (0.5%)	1 (0.5%)	0	0
Penicillin–streptomycin	1 (0.5%)	1 (0.5%)	0	0
Ampicillin	0	0	0	0
Piperacillin + tazobactam	0	0	0	0
Doxycycline	0	0	0	0
Total number of responses	307 (100%)	181 (100%)	75 (100%)	50 (100%)

SC, small clinics; LC, large clinics; VH, veterinary hospitals.

**Figure 3:**
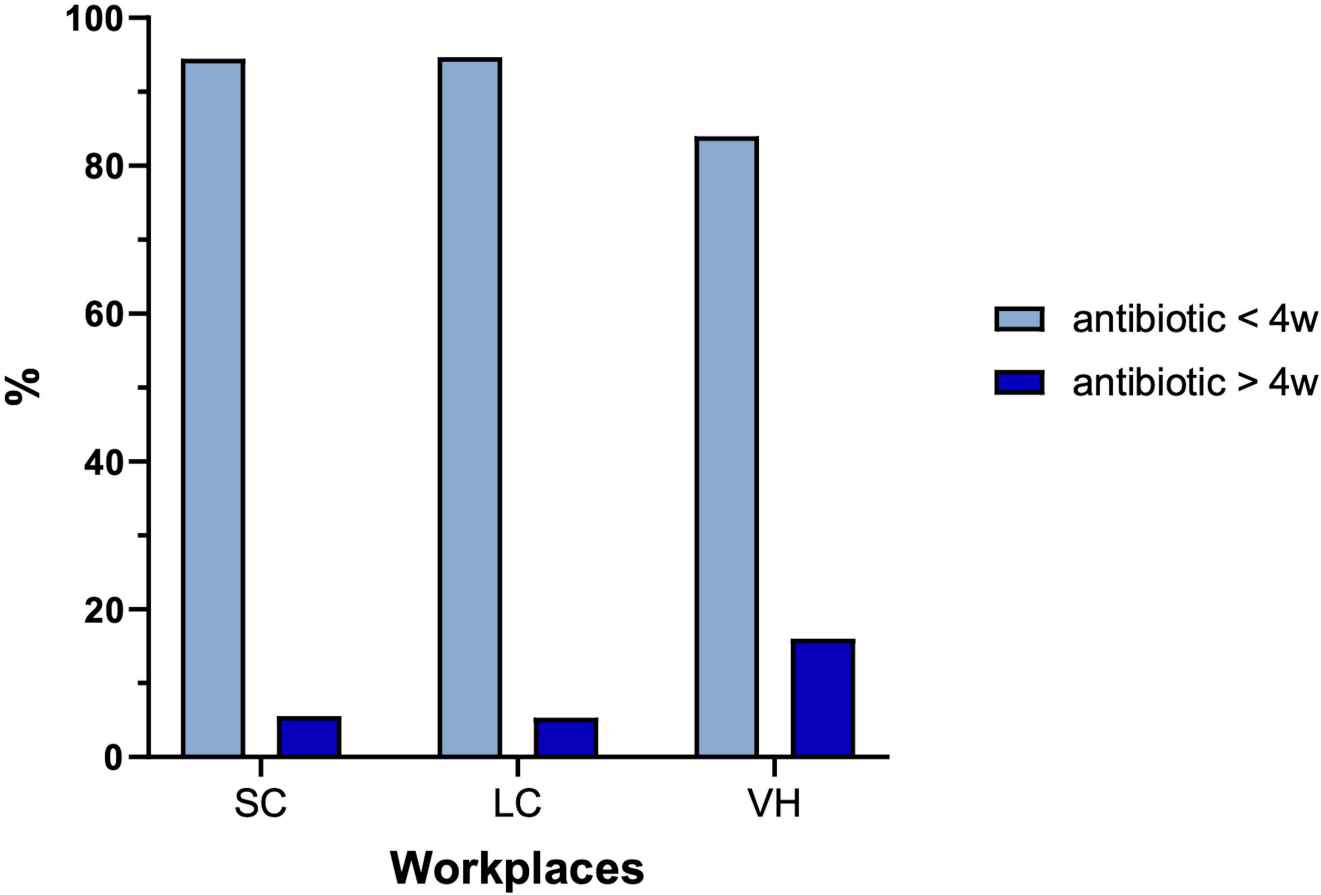
Percentage of responders treating dogs with acute prostatitis with more and less than 4 weeks of antibiotics in different workplaces. SC, small clinics; LC, large clinics; VH, veterinary hospitals.

The duration of antibiotic therapy is ≤ 2 weeks in 53.9% of cases, 2–4 weeks in 35.3%, 4–6 weeks in 7.2%, and not administered in 3.6% of cases. The proportion of veterinarians prescribing antibiotic therapy for more than 4 weeks significantly varies between types of veterinary facilities (*P* = 0.03; Figure 3).

Orchiectomy is performed by 180 (58.8%) veterinarians in fewer than 25% of cases, by 66 (21.6%) in 25%−50% of cases, by 36 (11.8%) in 50%−75% of cases, and by 24 (7.8%) in more than 75% of cases.

Hormonal therapy is selected by colleagues for various reasons (Table 4). The different drugs chosen are listed in Table 5. The therapeutic adjustments made by colleagues in cases where concurrent cysts larger than 2 cm were present are summarized in Table 6.

**Table 4:** Choice of hormonal treatment.

Possible answers (one or more)	Total number (%)	SC number (%)	LC number (%)	VH number (%)
It is my first choice; I only consider the surgical option in case of recurrence.	155 (40.5%)	110 (49%)	31 (33%)	14 (21%)
I choose it for patients whose owners do not want to proceed with castration (for ethical reasons, interest in maintaining the dog's fertility, behavioral issues, etc.).	105 (27%)	53 (24%)	27 (28%)	25 (38%)
I always perform it while waiting to schedule the orchiectomy procedure.	71 (18.5%)	34 (15%)	26 (27%)	11 (17%)
I choose it for patients who cannot undergo anesthesia.	46 (12%)	25 (11%)	10 (11%)	11 (17%)
I have never used it	8 (2%)	2 (1%)	1 (1%)	5 (7%)
Total number of responses	385 (100%)	224 (100%)	95 (100%)	66 (100%)

SC, small clinics; LC, large clinics; VH, veterinary hospitals.

**Table 5:** First-choice hormonal treatment.

Therapy	Total number (%)	SC number (%)	LC number (%)	VH number (%)
Osaterone acetate	217 (71%)	130 (72%)	57 (76%)	30 (60%)
Subcutaneous deslorelin implant	57 (18%)	37 (20%)	13 (18%)	7 (14%)
Finasteride	21 (7%)	12 (7%)	3 (4%)	6 (12%)
I do not use hormonal therapy	11 (4%)	2 (1%)	2 (2%)	7 (14%)
Total number of responses	306 (100%)	181 (100%)	75 (100%)	50 (100%)

SC, small clinics; LC, large clinics; VH, veterinary hospital.

**Table 6:** Is the treatment protocol modified when prostatic cysts exceed 2 cm in diameter?

Possible answers (one or more)	Total number (%)	SC number (%)	LC number (%)	VH number (%)
No	96 (31%)	69 (38%)	18 (24%)	9 (18%)
Yes, I perform ultrasound-guided aspiration of the cysts as a first approach	61 (20%)	25 (14%)	19 (25%)	17 (34%)
Yes, I combine the antibiotic with hormone therapy	45 (15%)	32 (18%)	8 (11%)	5 (10%)
Yes, I always associate orcheictomy with medical therapy	46 (16%)	32 (18%)	10 (13%)	4 (8%)
Yes, I perform cyst omentalization surgery	32 (10%)	11 (6%)	12 (16%)	9 (18%)
Yes, increased duration of antibiotic therapy	13 (4%)	5 (2%)	5 (7%)	3 (6%)
Other	13 (4%)	7 (4%)	3 (4%)	3 (6%)
Total number of responses	306 (100%)	181 (100%)	75 (100%)	50 (100%)

SC, small clinics; LC, large clinics; VH, veterinary hospitals.

### Follow-up

CST testing is performed at the end of the antibiotic therapy by 244 (79.7) veterinarians in fewer than 25% of cases, by 25 (8.2%) in 25%−50% of cases, by 13 (4.2%) in 50%−75% of cases, and by 24 (7.8%) in more than 75% of cases.

Recurrence of the condition within 6 months following treatment has been reported by 205 (67%) veterinarians in fewer than 25% of cases, by 77 (25.2%) in 25%−50% of cases, by 19 (6.2%) in 50%−75% of cases, and by 5 (1.6%) in more than 75% of cases.

The management strategies adopted in the event of recurrence are presented in Table 7.

**Table 7:** Approach to treating a possible recurrence.

Possible answers (one or more)	Total number (%)	SC number (%)	LC number (%)	VH number (%)
I propose orchiectomy if not yet performed	218 (48%)	123 (50%)	57 (49%)	38 (43%)
I propose prostatic omentalization surgery if cysts/abscesses are present	89 (20%)	34 (14%)	31 (26%)	24 (27%)
I add hormone therapy if not performed previously	55 (12%)	32 (13%)	11 (9%)	12 (14%)
I repeat the therapeutic protocol performed previously	48 (11%)	32 (13%)	9 (8%)	7 (8%)
Prolonged antibiotic therapy compared to previous therapy	22 (5%)	17 (7%)	3 (3%)	2 (2%)
Other^*^	17 (4%)	6 (3%)	6 (5%)	5 (6%)
Total number of responses	449 (100%)	244 (100%)	117 (100%)	88 (100%)

^*^Possible actions: performance of a urine culture and sensitivity tests not previously performed; complete re-evaluation, including blood biochemistry, urine culture and sensitivity tests, ultrasound with case reassessment; addition of supplements, vitamins, and probiotics.

SC, small clinics; LC, large clinics; VH, veterinary hospitals.

## Discussion

This study provides insight into the approach to acute prostatitis in dogs from the perspective of veterinary practitioners involved in small animal clinical practice. The initial diagnostic work-up for identifying prostatitis is consistent among most questionnaire respondents (93%), who, in accordance with the existing literature (7), diagnose acute prostatitis based on clinical signs, hematobiochemical analyses, urinalysis, and abdominal ultrasonography.

When a more specialized and in-depth diagnostic approach is required, the proportion of veterinarians performing additional testing decreases. In our study, only 18% of veterinarians reported using CPSE as part of the diagnostic work-up for prostatitis. Interestingly, no statistically significant difference was found between types of clinical facilities and the use of CPSE. The lack of a significant difference between types of clinical facilities in the use of CPSE suggests that the knowledge and application of this marker remain generally low across all settings, regardless of whether the facility provides basic care or 24-h care.

CPSE currently represents a promising non-invasive tool for the diagnosis of prostatic disorders in dogs. However, it is a non-specific marker: specific cut-off values for distinguishing between benign prostatic hyperplasia, prostatitis, or prostatic carcinoma have not yet been clearly established. It remains a general marker of prostatic damage, recently introduced into clinical practice, and its broader application will depend on further research (8, 23).

In our study, 24.2% of veterinarians reported never performing CST, considering it unnecessary, while 55% perform it in less than 25% of cases. The use of CST for the diagnosis of prostatitis varies significantly across different types of clinical facilities: it is performed in more than 50% of cases in only 16% of SC compared to 70% in VH. This discrepancy might initially reflect differences in antimicrobial stewardship, with larger facilities (often staffed by multiple clinicians) more likely to follow stricter clinical guidelines. Additionally, the variation could be influenced by differences in type of clientele, as clients attending 24-h hospitals may be more willing to invest in advanced diagnostic procedures than those visiting veterinary practices (24).

CST are essential to identify the causative agent(s) involved in the pathogenesis of prostatitis and to establish an appropriate therapeutic strategy (13). In human medicine, the classical gold standard for diagnosing chronic bacterial prostatitis is the collection of prostatic material via aspiration or expressed prostatic secretions, although both seminal fluid and post-massage urine have been shown to be more sensitive than standard urine samples in detecting prostatic infection (25). In canine patients, evidence remains conflicting regarding the optimal choice between prostatic-origin samples and urine collected via sterile catheterization (11). In our study, only 20% of veterinarians reported using prostatic-origin material for CST, while the majority (45%) preferred urine collected via sterile catheterization. According to the literature derived from human andrology, this approach may result in failure to diagnose bacterial prostatitis or incorrect identification of the causative agent, potentially leading to inappropriate antibiotic therapy. The International Society for Companion Animal Infectious Diseases (ISCAID) guidelines for canine patients recommend prostatic-origin material as the first-choice sample, with urine collected via sterile catheterization suggested only as a second-line option when prostatic sampling is not feasible (13).

Urine collection is technically easier than obtaining prostatic material. Semen collection is not always feasible, particularly in painful conditions such as acute prostatitis. As an alternative, prostatic fine-needle aspiration can be performed under sedation. When these procedures are not possible, sterilely collected urine remains a valid option, although its diagnostic limitations should be considered (11). The difficulty in obtaining prostatic-origin samples is indeed reported by veterinarians as one of the main reasons for not performing CST. The most frequently reported reasons for not submitting CST in our questionnaire are the owner's financial limitations and the extended turnaround time for laboratory results.

In our study the most selected empirical antibiotic was amoxicillin-clavulanic acid, chosen by 42% of respondents, followed by enrofloxacin (32%) and marbofloxacin (12%). The choice of empirical antibiotic significantly varied among different types of veterinary facilities. The implementation of an appropriate antimicrobial therapy is another critical aspect in the management of bacterial prostatitis. A key factor for therapeutic success is the use of antibiotics capable of penetrating the prostatic parenchyma, which typically requires lipid-soluble molecules. The ISCAID guidelines identify fluoroquinolones as the first-line empirical treatment while awaiting culture and susceptibility results, due to their favorable pharmacokinetic profile for prostatic penetration (13). According to the ISCAID guidelines, the recommended antibiotics in order of preference (pending culture results) include enrofloxacin, marbofloxacin, and trimethoprim-sulfamethoxazole. Other antibiotics may be considered based on susceptibility and pharmacological compatibility with the prostatic environment (13).

Our findings indicate that the duration of antibiotic therapy was ≤ 2 weeks in 53.9% of cases and 2–4 weeks in 35.3%. Treatment duration is another essential component of effective prostatitis management. The ISCAID guidelines recommend a minimum duration of 4 weeks, with possible extension based on clinical response and follow-up diagnostics (13). The proportion of veterinarians prescribing antibiotic therapy for more than 4 weeks significantly varied by practice type. Although approximately 80% of practitioners do not adhere to the recommended treatment duration outlined in current guidelines, VH appear to show slightly better compliance. This could be attributed to the presence of specialized departments—such as reproductive medicine or andrology—with more experienced personnel and updated clinical training. These differences could also reflect disparities in antimicrobial stewardship and continuing education, particularly in smaller clinics, where limited staffing and the absence of internal clinical protocols could influence prescribing behavior. Additionally, client-related factors may impact decisions: owners attending outpatient or general practices may be less inclined or able to commit to prolonged treatments exceeding 2 weeks. Another important consideration is that current regulations restrict the use of fluoroquinolones (e.g., enrofloxacin or marbofloxacin) to cases supported by CST, and ideally, this should discourage their empirical use. However, in everyday clinical practice, this is not always feasible, and fluoroquinolones are still sometimes prescribed without prior CST, reflecting practical constraints and clinical decision-making in the absence of diagnostic confirmation.

In our study, 19.6% of veterinarians chose orchiectomy within 1 month of the diagnosis of acute prostatitis in more than 50% of cases. Orchiectomy significantly reduces the likelihood of recurrence by eliminating testosterone-induced hormonal stimulation of the prostate, and it can be considered in patients where the owner is not interested in preserving fertility, concomitant testicular pathologies are present (e.g., tumors), and the anesthetic risk is acceptable (i.e., no renal impairment) (26). In 50.7% of cases, the veterinarians in our study chose hormonal medical therapy as the first-line treatment and considered orchiectomy only in cases of recurrence. It was chosen less frequently in dogs that could not undergo orchiectomy for various reasons (such as anesthetic risk or the need to preserve fertility), or while awaiting the surgical procedure. The most selected therapy by the colleagues was osaterone acetate, followed by the subcutaneous deslorelin implant. Treatment with osaterone acetate allows a rapid reduction of hormonal stimulation on the prostate within a few days, by targeting the underlying benign prostatic hyperplasia that contributes to prostatic inflammation and thus may support the medical management of acute prostatitis (27). Deslorelin may not be suitable in acute conditions, as its effect begins 6–8 weeks after implantation, and it may initially cause a rebound effect that worsens the clinical picture (27, 28).

Only 7.8% of cases opted to repeat the CST at the end of antibiotic therapy. Currently, there are no established guidelines indicating whether and how to initiate treatment in cases of positive CST results in asymptomatic dogs showing no abnormalities in bloodwork, urinalysis, or fertility. It is therefore unclear whether antibiotic therapy should aim for complete eradication of the pathogen from the organ, or simply for remission of clinical signs and normalization of laboratory parameters. As such, no clear recommendation exists regarding the need to repeat the CST.

Regarding the management of potential recurrences, therapeutic approaches reported by colleagues vary. Recommendations include considering castration, either surgical or chemical (if not already performed), repeating the CST to assess the development of antimicrobial resistance, and performing more in-depth diagnostics, such as cytological examination (13). Similarly, the approach to prostatic cysts was not consistent among the responses collected in our study. In cases where prostatic cysts larger than 2 cm are present, the literature supports ultrasound-guided aspiration, and in the event of recurrence, surgical omentalization is advised (2).

This study has some limitations. First, the study was based on voluntarily provided responses, which could not accurately reflect the participants' actual clinical practices. Asking explicitly how a given situation would be managed may lead respondents to indicate what they believe is the correct or expected answer, rather than what they truly do in practice. Second, individual clinicians may apply different diagnostic and therapeutic approaches depending on case-specific factors not evaluated in this survey, such as disease severity, comorbidities, or owner-related constraints, contributing to intra-observer variability. Third, we did not collect information on respondents' years of practice or the number of acute prostatitis cases managed per month, as the questionnaire was intentionally kept short to facilitate rapid completion. This limits the assessment of respondents' clinical experience and caseload, which should be considered when interpreting and generalizing the findings. Fourth, as no formal definition of acute prostatitis was provided in the questionnaire, respondents may have interpreted this condition differently, potentially contributing to response variability. Fifth, another limitation is the heterogeneity of the respondent groups, with a predominance of participants working in SC, which may affect the generalizability of the findings. Finally, the study was designed as a preliminary exploratory survey with a limited sample size. This precluded the use of multivariable statistical models, and no correction for multiple comparisons was applied. Consequently, the findings are purely descriptive, and causal or inferential conclusions cannot be drawn.

In conclusion, this study offers an overview of how acute prostatitis in dogs is approached by small animal practitioners, highlighting both strengths and critical gaps in diagnostic and therapeutic practices. While the initial diagnostic work-up is largely consistent with current recommendations, more advanced diagnostics such as CPSE measurement and CST are underutilized, often due to practical limitations like cost and turnaround times. The use of empirical antibiotic therapy frequently deviates from ISCAID guidelines, particularly in terms of antibiotic selection and treatment duration, raising concerns about antimicrobial stewardship. Moreover, the low adoption of prostatic-origin sampling may hinder accurate diagnosis and appropriate therapy. Differences in diagnostic and therapeutic strategies between types of facilities reflect disparities in resources, clinical training, and client expectations. These findings underscore the need for continued professional education, wider dissemination of evidence-based guidelines, and greater emphasis on diagnostic standardization to improve patient outcomes in canine prostatitis.

## Data Availability

The raw data supporting the conclusions of this article will be made available by the authors, without undue reservation.
