# Supplementary material for: Diagnostic and therapeutic approaches to acute prostatitis in dogs: a survey of Italian veterinary practitioners
Source: Front Vet Sci. 2026 Apr 17;13:1774785. doi: 10.3389/fvets.2026.1774785 (PMC13132774; doi:10.3389/fvets.2026.1774785)
Supplement: Supplementary file 1 [file Data_Sheet_1.docx]

SURVEY: HOW DO YOU TREAT ACUTE PROSTATITIS IN DOGS?

- In which region do you work?
- In which province do you work?
- In which type of facility do you work?
- Veterinary practice
- Veterinary clinic
- Veterinary hospital (24/7)
- How do you diagnose acute prostatitis in dogs?
- Clinical signs
- Clinical signs + blood tests
- Clinical signs + urinalysis
- Clinical signs + blood tests + urinalysis
- Clinical signs + blood tests + urinalysis + abdominal ultrasound
- Do you request serum CPSE dosage to make the diagnosis?
- Yes
- No
- Do you perform bacterial culture + antibiotic sensitivity testing?
- In more than 75% of cases
- In 50–75% of cases
- In 25–50% of cases
- In less than 25% of cases
- On which samples do you perform culture + sensitivity testing?
- Urine collected by sterile catheterization
- Urine collected by cystocentesis
- Urine collected by natural urination
- Prostatic fraction of ejaculate
- Prostatic and/or cyst aspirate if present
- Prostatic fluid collected by catheter and prostatic massage
- I do not perform the test
- What are the most frequent reasons for not performing culture + sensitivity testing? (Choose up to two)
- Owner's financial constraints
- Difficulty in sample collection
- Difficulty sending sample to diagnostic lab
- Delayed results
- I don't consider it necessary
- How do you usually treat prostatitis in dogs?
- Antibiotic
- Anti-inflammatory
- Hormonal therapy
- Antibiotic + hormonal therapy
- Antibiotic + anti-inflammatory + hormonal therapy
- Other

*Hormonal therapy refers to: Osaterone Acetate, Finasteride, subcutaneous Deslorelin implant

- What percentage of dogs diagnosed with acute prostatitis undergo orchiectomy within one month of diagnosis?
- In more than 75% of cases
- In 50–75% of cases
- In 25–50% of cases
- In less than 25% of cases
- Hormonal therapy (choose up to two options):
- I choose it for patients that cannot undergo anesthesia
- I choose it when owners do not want castration (ethical reasons, desire to maintain fertility, behavioral concerns, etc.)
- I always use it while awaiting orchiectomy
- It is my first choice; I consider surgery only if there is a relapse
- I have never used it
- Which drug do you normally prescribe for hormonal therapy?
- Osaterone acetate
- Finasteride
- Subcutaneous Deslorelin implant
- I do not use hormonal therapy
- Which antibiotic do you empirically prescribe (while awaiting or without sensitivity test)?
- Enrofloxacin
- Marbofloxacin
- Amoxicillin + clavulanic acid
- Ampicillin-sulbactam
- Ampicillin
- Doxycycline
- Trimethoprim-sulfa
- Clindamycin
- Cephalexin
- Ceftriaxone
- Cefovecin
- Metronidazole
- Piperacillin + tazobactam
- I do not use antibiotics
- What is the average duration of antibiotic therapy you prescribe?
- ≤ 2 weeks
- 2–4 weeks
- 4–6 weeks
- 6–8 weeks
- > 8 weeks
- Do you modify your therapeutic protocol in the presence of prostatic cysts > 2 cm?
- No
- Yes, I perform ultrasound-guided aspiration as a first approach
- Yes, I increase the duration of antibiotic therapy
- Yes, I perform cyst omentalization surgery along with orchiectomy as first approach
- Yes, I add hormonal therapy to antibiotics
- Other
- Do you perform bacterial culture + sensitivity test at the end of antibiotic therapy?
- In more than 75% of cases
- In 50–75% of cases
- In 25–50% of cases
- In less than 25% of cases
- How many dogs with prostatitis experience a relapse within 6 months of treatment?
- More than 75% of cases
- 50–75% of cases
- 25–50% of cases
- Less than 25% of cases
- How do you manage a possible relapse?
- I propose orchiectomy if not already done
- I extend the antibiotic therapy compared to the previous one
- I add hormonal therapy if not previously used
- I propose cyst/abscess omentalization surgery
- I repeat the previous treatment protocol
- Other
