# Supplementary material for: Diagnostic and therapeutic approaches to acute prostatitis in dogs: a survey of Italian veterinary practitioners
Source: Front Vet Sci. 2026 Apr 17;13:1774785. doi: 10.3389/fvets.2026.1774785 (PMC13132774; doi:10.3389/fvets.2026.1774785)
Supplement: Supplementary file 2 [file Data_Sheet_2.docx]

S2 - Number of Responses per Region

| **Region** | **Number of Responses** |
| --- | --- |
| Abruzzo | 6 |
| Calabria | 6 |
| Campania | 4 |
| Emilia Romagna | 61 |
| Friuli Venezia Giulia | 6 |
| Lazio | 20 |
| Lombardia | 64 |
| Marche | 18 |
| Molise | 1 |
| Piemonte | 35 |
| Sardegna | 2 |
| Toscana | 50 |
| Trentino Alto Adige | 5 |
| Umbria | 7 |
| Val D’Aosta | 1 |
| Veneto | 20 |
